# New locus reveals the genetic architecture of sex reversal in the Chinese tongue sole (*Cynoglossus semilaevis*)

**DOI:** 10.1038/s41437-018-0126-6

**Published:** 2018-08-09

**Authors:** Yu Cui, Weifeng Wang, Liyong Ma, Jinhua Jie, Yanhong Zhang, Huanling Wang, Hengde Li

**Affiliations:** 1 National Demonstration Center for Experimental Fisheries Science Education, Shanghai Ocean University, Shanghai, 201306 China; 2 Centre for Applied Aquatic Genomics, Chinese Academy of Fishery Sciences, Beijing, 100141 China; 3 College of Fisheries, Huazhong Agricultural University, Wuhan, 430070 China; 4 Hanhai Aqua Co. Ltd. Weifang, 261311 Shangdong Province, China; 5 Jiehai Aqua Co. Ltd. China-Czech High Tech Park., Cangzhou, 061108 China; 6 Key Laboratory of Aquatic Genomics, Ministry of Agriculture, CAFS Key Laboratory of Aquatic Genomics and Beijing Key Laboratory of Fishery Biotechnology, Chinese Academy of Fishery Sciences, Beijing, 100141 China

## Abstract

Sex reversal in insects, amphibians, reptiles, and fishes is a complicated and interesting biological phenomenon. Sex reversal changes the sex ratio of populations and may complicate breeding schemes. In the Chinese tongue sole (*Cynoglossus semilaevis*), genetic females may change into pseudomales, thereby increasing aquaculture costs because of the lower growth rate of the males than that of the females. Here we identify a new locus associated with sex reversal; this single nucleotide polymorphism (SNP) is located in the third intron of the doublesex and mab-3 related transcription factor 1 (*Dmrt1*) gene on the Z chromosome (named Cyn_Z_8564889) and has two alleles, A and G. Cyn_Z_8564889 regulates sex reversal interactively with our previously detected SNP (Cyn_Z_6676874), with the genetic females simultaneously carrying the T allele of Cyn_Z_6676874 and the A allele of Cyn_Z_8564889 changing into pseudomales. Other *Dmrt1* polymorphisms were detected, which formed two haplotypes. Two SNPs in the second exon of *Dmrt1* result in amino acid changes, suggesting that *Dmrt1* is essential in sex reversal. We also verified that pseudomales produce no or little W sperm. The interaction and linkage between Cyn_Z_6676874 and Cyn_Z_8564889 and the absence of W sperm from pseudomales unravel the genetic architecture of sex reversal in *C. semilaevis*.

## Introduction

The sex of vertebrates is usually determined by chromosomal form, called genetic sex determination (GSD), e.g., the XY male/XX female system in mammals and the ZW female/ZZ male system in birds. However, for some species, the primary sex can be changed during development. Sex reversal is an interesting biological phenomenon and can happen in insects (Narita et al. 2007), reptiles (Quinn et al. 2007), amphibians (Wallace et al. 1999), and fish (Nagahama 2005). Ecologists consider it an evolutionary reproductive strategy for animals to adapt to the environment (Ghiselin 1974; Shapiro 1987). Environmental factors, such as temperature, hormones, and pH, may affect sex reversal, but many studies have focused on temperature. High temperature can induce female-to-male sex reversal in *Triturus cristatus* (Wallace et al. 1999) and the Chinese tongue sole (*Cynoglossus semilaevis*) (Chen et al. 2014) and male-to-female reversal in *Pogona vitticeps* (Holleley et al. 2015). Environment-dependent sex determination (ESD) and GSD are not mutually exclusive (Sarre et al 2004). It is widely acknowledged that the sex of vertebrates is determined by genetic factors, which can interact with external environmental factors in the early stages of development (Shapiro 1987). In *Oryzias latipes*, sex reversal is controlled simultaneously by an autosomal locus and sex-determining region on the Y chromosome (Kato et al. 2010, 2011; Shinomiya et al. 2005). In *P. vitticeps*, sex reversal can shift from GSD to temperature-dependent sex determination (TSD), indicating that genetic background may be critical (Jiang and Li 2017). Several genes have been found to be involved in TSD in turtles (Chojnowski and Braun 2012), showing the interaction between genetic and environmental factors.

Sex reversal changes the sex ratios in populations, which may affect broodstock management in fish farming. The Chinese tongue sole is an indigenous and important economic marine flatfish in China. Female fish are preferred in tongue sole aquaculture because the adult female is 2–4 times larger than the adult male. However, some genetic female tongue soles reverse into phenotypic males at day 50–90. Sex-reversed or pseudomale fish grow as slowly as normal males do. Because of sex reversal, the female percentages have decreased gradually in recent years and currently average below 20%. Sex reversal has increased costs considerably for farmers, and improving and maintaining female ratios have become urgent and important issues in tongue sole aquaculture. Some investigators have attempted to improve female ratios by producing all-female offspring with super female WW fish (Chen et al. 2012), but this is not feasible because WW fish cannot survive the early stages of development (Chen et al. 2014). Use of pseudomales was also considered to improve female ratios, but all genetic female offspring of pseudomales change to pseudomales. In our previous study (Jiang and Li 2017), single nucleotide polymorphism (SNP) Cyn_Z_6676874 was associated with sex reversal, and selecting male broodstock with Z^A^Z^A^ genotype improved the female ratio to 0.5. Although this SNP explains ~83% of genetic variance, it was obvious that other genes were involved in sex reversal because not all Z^T^W genetic females changed into pseudomales. In the current study, we sampled genetic females of only the Z^T^W genotype of SNP Cyn_Z_6676874, obtained their sex-reversal phenotype using tissue sections, and performed a genome-wide association study (GWAS) to detect the genetic loci interacting with the SNP Cyn_Z_6676874. A new locus was identified to regulate sex reversal interactively with the SNP Cyn_Z_6676874; the linkage between these two loci and the absence of W sperm for pseudomales clearly elucidates the genetic architecture of sex reversal in the tongue sole.

## Materials and methods

### Materials

*Population 1:* A batch of fry was randomly collected from nine families at Hanhai Aqua. Co. Ltd. at Weifang, Shandong Province, China, in 2016. The fry was raised for 120 days at a constant temperature of 22 °C in the same pond to avoid environmental effects. Five hundred fish were randomly selected for sex determination and tissue sections. Based on our previous study (Jiang and Li 2017), all fish were genotyped for the SNP Cyn_Z_6676874, from which 171 fish of the Z^T^W genotype were selected for 2b-restriction site–associated DNA (RAD) genotyping and GWAS. Among these fish, 94 changed into males, and 77 were normal females.

*Population 2:* In August 2017, 500 fish that were unknown to be closely related to population 1 were randomly harvested from Jiehai Aqua Co. Ltd. Among them, 259 genetic females were identified and used for validation of our newly detected polymorphism associated with sex reversal, and the genetic interaction between it and the SNP Cyn_Z_6676874.

*Population 3:* It has been reported that pseudomale tongue sole will not produce W spermatozoa (Holleley et al. 2015). This finding is critically important for us to draw conclusions about the genetic architecture of the sex reversal of tongue sole, because it is difficult to prevent somatic cells from contaminating the milt during squeezing. In order to examine whether a pseudomale will produce W spermatozoa, 15 mature pseudomales were randomly selected from Jiehai Aqua. Co. Ltd. Their fins and semen were collected for DNA extraction, representing somatic cell DNA and sperm cell DNA, respectively.

### Phenotype

Fins and gonads were sampled for DNA extraction and sex determination. The genetic sex was determined with fin DNA using a previously described sex-specific marker (Liu et al. 2014). Phenotypic sex was determined by tissue sectioning of gonads; the details of the tissue sectioning were described in our previous study (Jiang and Li 2017). Sex reversal was determined by comparing genetic and phenotypic sex, accordingly.

### DNA extraction

DNA was extracted from the fins of all individuals. To verify whether a pseudomale can produce W sperm, DNA was also extracted from the semen of the pseudomales. The DNA extraction protocols for fins and semen were similar and as follows:

(1) Semen DNA preparation: Frozen semen (~3 mg) and 200 μl lysis buffer was placed in a 1.5 ml centrifuge tube and vortexed for 10 min at room temperature(20–25 °C) The mixture was centrifuged at 12,000 r.p.m. for 1 min and the supernatant was discarded. Lysis buffer (170 μl) and 5 μl of proteinase K (20 mg/ml) were added to the pellet, mixed thoroughly by vortexing, and then digested at 65 °C for 2 h. Five microliters of DNase-I (5 U/μl) and 20 μl 10× reaction buffer was added, and the mixture was digested at 37 °C for 30 min. The mixture was centrifuged at 3000 r.p.m. for 15 min. One milliliter of ethanol was added to the supernatant and the precipitate was pelleted by centrifugation at 12,000 r.p.m. for 1 min. The DNA pellet was dried at room temperature for 20 min.

(2) Fin DNA preparation: Approximately 10 mg of fin was minced or ground and placed in a 1.5 ml centrifuge tube. Lysis buffer (400 μl) and 5 μl proteinase K (20 mg/ml) was added and the mixture incubated at 65 °C until the insoluble material was completely dissolved. The mixture was extracted with an equal volume of phenol: chloroform: isoamyl alcohol (25: 24: 1), and the aqueous phase was transferred to a new 1.5 ml centrifuge tube. After adding an equal volume of isopropanol and 0.1 volume of 3 M sodium acetate (pH = 5.2), the mixture was centrifuged at 12,000 r.p.m. for 10 min and the supernatant discarded. Finally, the pellet was washed three times with 1 ml 75% ethanol, centrifuging at 12,000 r.p.m. for 2 min between washes. The DNA pellet was dried at room temperature for 20 min.

All dried pellets were dissolved in 20 μl sterilized water and stored at 4 °C.

### Genotypes

A total of 1071 genetic females in population 1 were genotyped using the 2b-RAD method from Oebiotech Co. Ltd. (Shanghai, China). The 2b-RAD libraries were prepared with *Bsa*XI following the reported protocol (Wang et al. 2012), and subjected to single-end sequencing using an Illumina Hiseq 2500 platform. By sequencing, a total of 1,807,470,684 reads was produced, averaging 10,570,004 reads per sample. The 2b-RAD genotyping was performed with RADtyping v1.5 software (Fu et al. 2013). Reads with no restriction sites, or those containing ambiguous base calls (N), long homopolymer regions (>10 bp), or excessive numbers of low quality positions (>10 positions with quality of <20) were removed. Finally, 186,928 unique tags were obtained; the average level of coverage was 37× per sample. In total, 136,267 SNP genotypes were obtained and mapped to the *C. semilaevis* genome with SOAP2 (Li et al. 2009). The SNPs with minor allele frequencies of <5% or call rates of <0.80 were discarded. The missing genotypes were imputed through disequilibrium with 10 close neighboring markers, as in our previous study (Jiang and Li 2017). After quality control, the final genotypic data in the analysis consisted of 33,410 SNPs without missing genotypes (Table 1).

**Table 1:** Single nucleotide polymorphism (SNP) information

Chromosome	Number of SNPs	Span of SNPs (Mb)	Distance between adjacent markers (kb)
1	2961	34.50733	11.65788
2	1753	20.04642	11.44202
3	1372	16.21593	11.82781
4	1597	19.96116	12.50699
5	1734	19.26109	11.1143
6	1544	18.82236	12.19855
7	1309	13.78154	10.53635
8	2395	30.12243	12.58247
9	1597	19.59837	12.27968
10	1644	20.95554	12.75444
11	1612	20.46002	12.7002
12	1556	18.35167	11.80172
13	1576	21.85925	13.87889
14	1951	28.83096	14.78511
15	1503	20.05586	13.35277
16	1491	18.77584	12.60123
17	1414	16.45747	11.64718
18	1236	15.0991	12.22599
19	1345	17.72299	13.18675
20	1215	15.20323	12.52325
W	119	15.83972	134.2349
Z	486	21.14982	43.60788
Total	33410	443.0781	19.33847

### Statistical analysis

Sex reversal was considered a binomial trait, and the incidence of sex reversal was recorded as 1, while its absence was recorded as 0. A logistic linear mixed model was used to perform GWAS:

1 $${{\mathbf{logit}}}\left( y \right) = \mu + bm + g + e,$$

where logit *(y)* = *p*/(1-*p*), and *p* is the frequency of sex reversal, *m* is the SNP genotype (assumed to be a fixed effect), *b* is the allele substitution effect, *g* is the additive effect that follows the distribution N(0, **K***σ*_*g*_^2^), **K** is the realized genetic relationship matrix, and e is the random error that follows the distribution N(0, **K***σ*_*g*_^2^). Construction of **K** followed these procedures: (1) code the genotype matrix **M** for each marker, 0 for homozygote, 1 for heterozygote, and 2 for the other homozygote, (2) standardize **M** to **Z** with the mean and the standard deviation of each SNP, and (3) calculate **K** as **ZZʹ**/*n*_*m*_, where *n*_*m*_ is the number of markers. An association analysis of each SNP was conducted by comparing the full model to the null model:

2 $${\it{{\rm logit}}}\left( y \right) = \mu + g + e,$$

The −2*log-likelihood test statistics approximately followed a *χ*^2^ distribution with a degree of freedom of 1. To improve the computational speed, a method similar to that of the efficient multi-locus mixed model (Segura et al. 2012) was adopted. First, DMU (Madsen and Jensen 2002) was employed to estimate the variance components, *σ*_*g*_^2^ and *σ*_*e*_^2^. Then, the linear mixed model was transformed to a simple linear regression through the Cholesky decomposition of the phenotypic covariance structure **V** as the following equation:

3 $$y^\ast = bm^\ast + e^\ast,$$

where y* = **V**^−^^1/2^ logit*(y)*, M* = **V**^−1/2^**M**, **V** **=** **K***σ*_*g*_^2^ + **I***σ*_*e*_^2^, and m* is the corresponding SNP column of M* matrix. After a genome san, whether a quantitative trait locus (QTL) existed was determined according to the critical threshold value calculated with Peipho’s approach (Piepho 2001). Once a genetic locus was identified, the corresponding 95% confidence interval was calculated using Li’s method (Li 2011).

### Ethics statement

All the handling of fish was conducted in accordance with the Guidelines on the Care and Use of Animals for Scientific Purposes setup by the Chinese Academy of Fishery Sciences.

## Results

### Genome-wide association study

**Fig. 1:**
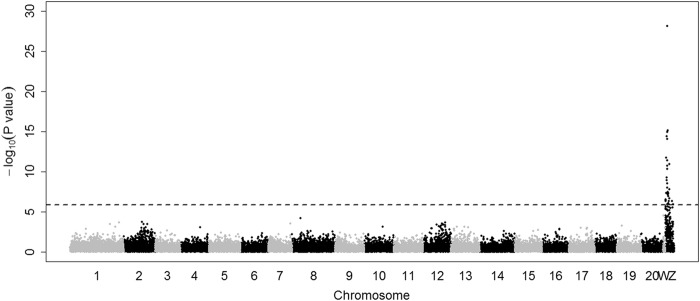
Genome-wide association study (GWAS) of sex reversal of half-smooth tongue sole of Z^T^W genotype on SNP Cyn_Z_6676874. The *x*-axis represents the genomic coordinates along chromosomes 1–20 and sex chromosomes W and Z. The *y*-axis represents the negative logarithm of *P*-values. The horizontal dashed line is the genome-wide threshold; SNPs above this line are significantly associated with sex reversal

GWAS was conducted with individuals of genotype Z^T^W of the SNP Cyn_Z_6676874. A single SNP at 8,564,889 bp on the Z chromosome (named Cyn_Z_8564889) was found to be strongly associated with sex reversal in the Half-smooth tongue sole (*P* < 1.0 × 10^−^^28^, Fig. 1) of genotype Z^T^W at the SNP Cyn_Z_6676874. The new SNP was located at the third intron of the doublesex and mab-3 related transcription factor 1 (*Dmrt1*) gene, which is known to be gonad-specific and shows sexually dimorphic expression patterns. The protein that it codes contains a conservative base sequence with DNA binding capacity, named DM structural domain (doublesex and Mab-3). The zinc finger structure combined with specific DNA sequences participates in the regulation of sex determination and gonadal differentiation in vertebrates and non-vertebrates (Smith et al. 1999). SNP Cyn_Z_8564889 has two alleles, G and A, and there are usually two genotypes for pseudomales, because it is located on the Z chromosome. However, two individuals in population 1 were genotyped as AG by sequencing PCR products (Fig. S1), which showed the possibility of duplication of *Dmrt1* in Chinese tongue sole. The individuals with Z^T,GG^W genotype (the superscript corresponds to the genotypes of Cyn_Z_6676874 and Cyn_Z_8564889, respectively) would not reverse; whereas, the individuals with Z^T,AG^W and Z^T,AA^W genotypes all reversed into phenotypic males. The 95% confidence interval of the SNP Cyn_Z_8564889 was from 8,564,250 to 8,610,045 bp, where only two known genes were located. These two genes were *Dmrt1* and *Kank1* (KN motif and ankyrin repeat domains 1), the latter of which is involved in the control of cytoskeletal formation by regulating actin polymerization.

### Haplotype in *Dmrt1*

**Fig. 2:**
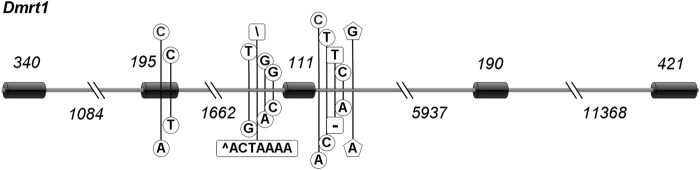
Structure of the *Dmrt1* gene and the polymorphisms that it contains. The horizontal cylinders and gray lines represent the exons and introns of *Dmrt1*, respectively, and the numbers indicate their corresponding lengths. The vertical lines show the positions of polymorphisms. The pentagon-surrounded bases are the two alleles of SNP Cyn_Z_8564889, the circled bases are the SNPs, and the orthogon-surrounded bases are indels. The polymorphisms formed two haplotypes, the upper and lower polymorphisms stand for each haplotypes, respectively

Alignment of *Dmrt1* cDNA sequences (NCBI Accession: EU007655) to Z chromosome genomic sequences of tongue sole (NCBI Accession: NC_024328.1) determined that *Dmrt1* contains five exons and four introns. The fragments amplified for genotyping SNP Cyn_Z_8564889 contained partial sequences of the second and third intron of *Dmrt1*. Besides SNP Cyn_Z_8564889, six SNPs and two indels were found in this region (Fig. 2, Table 2), and the indel in the second intron of *Dmrt1* is a 7-bp insertion/deletion polymorphism. To explore whether there are sex-related SNPs on other coding sequence (CDS) regions of *Dmrt1*, primers were designed and the other four *Dmrt1* exons were amplified (Table 3). Two SNPs were found in the second exon (Fig. 2, Table 2); the SNP C → A at position 8,566,940 and SNP C → T at position 8,566,916 will cause the amino acid changes Ala → Ser and Val → Ile, respectively (Fig. S2). Therefore, we speculated that these amino acid changes affected the sex reversal of the Half-smooth tongue sole. By sequencing 15 fish of Z^T,GG^W genotype and 15 fish of Z^T,AA^W genotype, the nine SNPs and two indels that we found in *Dmrt1* formed two haplotypes (Fig. 2, Table 2). These polymorphisms can also be used to genotype the SNP Cyn_Z_8564889 as a substitution.

**Table 2:** Characteristics of the two haplotypes in *Dmrt1*

Allele in Haplotype 1	Allele in Haplotype 2	Region	Position
A	C	Exon 2	8566940
T	C	Exon 2	8566916
G	T	Intron 2	8565228
ACTAAAA	/	Intron 2	8565219
A	G	Intron 2	8565217
C	G	Intron 2	8565187
C	T	Intron 3	8565031
A	C	Intron 3	8565018
/	T	Intron 3	8565016
T	C	Intron 3	8565011
A	G	Intron 3	8564889

**Table 3:** Primers to amplify target fragments of *Dmrt1*

Primer name	Primer sequence (5′-3′)	Tm (℃)	Product length
Cyn_Z_8564889_PF	CAACTAAGACTTTTCAAACCC	62	643
Cyn_Z_8564889_PR	TTAAAATCCCAACACTCACAC	62	
Dmrt1_exon1_PF	CCATTAATTACAAAATGTTACAGG	60	494
Dmrt1_exon1_PR	AGTAGCAGTAGCAGTATCAGT	60	
Dmrt1_exon2_PF	TAAGGCAATAAAGAAAGGTAAGGTC	60	298
Dmrt1_exon2_PR	GCTGTGTTGTGAAGTCTGGT	60	
Dmrt1_exon3_PF	TGTCCTTCTATCACTGCATTCTCA	62	276
Dmrt1_exon3_PR	GTGTTTTCCAAATGTATCAGTATGC	62	
Dmrt1_exon4_PF	AACAGGTAACAAGTACATTTCTGTG	62	391
Dmrt1_exon4_PR	TGAATGTCGAACGAGCAGGG	62	
Dmrt1_exon5_PF	GGAGCGAGTCATTTGATCAGG	60	535
Dmrt1_exon5_PR	TGATTGGATCAGAGCATGATTGT	60	

PCR amplification was in a volume of 40 μl, containing 20 μl PCR Mix (TaKaRa), 50 ng template, and 0.5 μM of each primer. The PCR conditions were: 95 °C for 3 min, followed by 32 cycles of 94 °C for 30 s, corresponding melting temperature (Tm) for 30 s, 72 °C for 1 min, and a final extension step at 72 °C for 5 min

### Genetic interaction

It had been confirmed in our previous study that Z^A^W fish at SNP Cyn_Z_6676874 do not reverse into pseudomales (Jiang and Li 2017), but the new locus SNP Cyn_Z_8564889 was identified with only Z^T^W samples in this study. Therefore, it was obvious that there was a genetic interaction between SNP Cyn_Z_6676874 and Cyn_Z_8564889, which simultaneously regulated sex reversal in tongue sole. Genetic females will reverse into pseudomales only if they contain both allele T in SNP Cyn_Z_6676874 and allele A in SNP Cyn_Z_8564889 simultaneously (Table 4). These two SNPs explained all genetic variance of sex reversal in tongue sole.

**Table 4:** Interaction between the SNPs Cyn_Z_6676874 and Cyn_Z_8564889

Cyn_Z_8564889	Cyn_Z_6676874	
	A	T
GG	Female (91)	Female (123)
AA	Female (0)	Pseudomale (39)
AG	Female (1)	Pseudomale (5)

SNPs Cyn_Z_6676874 and Cyn_Z_8564889 loci interactively regulate sex reversal in the Chinese tongue sole; genetic female fish containing both the T allele of SNP Cyn_Z_6676874 and the A allele of SNP Cyn_Z_8564889 simultaneously reverse into pseudomales. This genetic interaction was confirmed. The number of fish with the genotypic combination of these two loci in population 2 is shown in parentheses

### Validation

To confirm SNP Cyn_Z_8564889 and its interaction with the SNP Cyn_Z_6676874, the target fragments containing these two SNPs were amplified and sequenced for population 2. Considering SNP Cyn_Z_6676874, among 259 genetic female fish (Table 4), 92 individuals were the Z^A^W genotype and none of them reversed into pseudomales, while 44 individuals of the Z^T^W genotype reversed into pseudomales. This was consistent with the results of our previous study (Jiang and Li 2017). Considering SNP Cyn_Z_8564889, 214 fish were the Z^GG^W genotype and none of them reversed into pseudomales, while 39 Z^AA^W and 5 Z^AG^W genotype fish reversed. Considering SNP Cyn_Z_6676874 and Cyn_Z_8564889 simultaneously, only the Z^T,A_^W genotype fish reversed, while the fish containing either the A allele of SNP Cyn_Z_6676874 or the GG genotype for SNP Cyn_Z_8564889 did not reverse into pseudomales (Table 4).

### Spermatogenesis of pseudomales

**Fig. 3:**
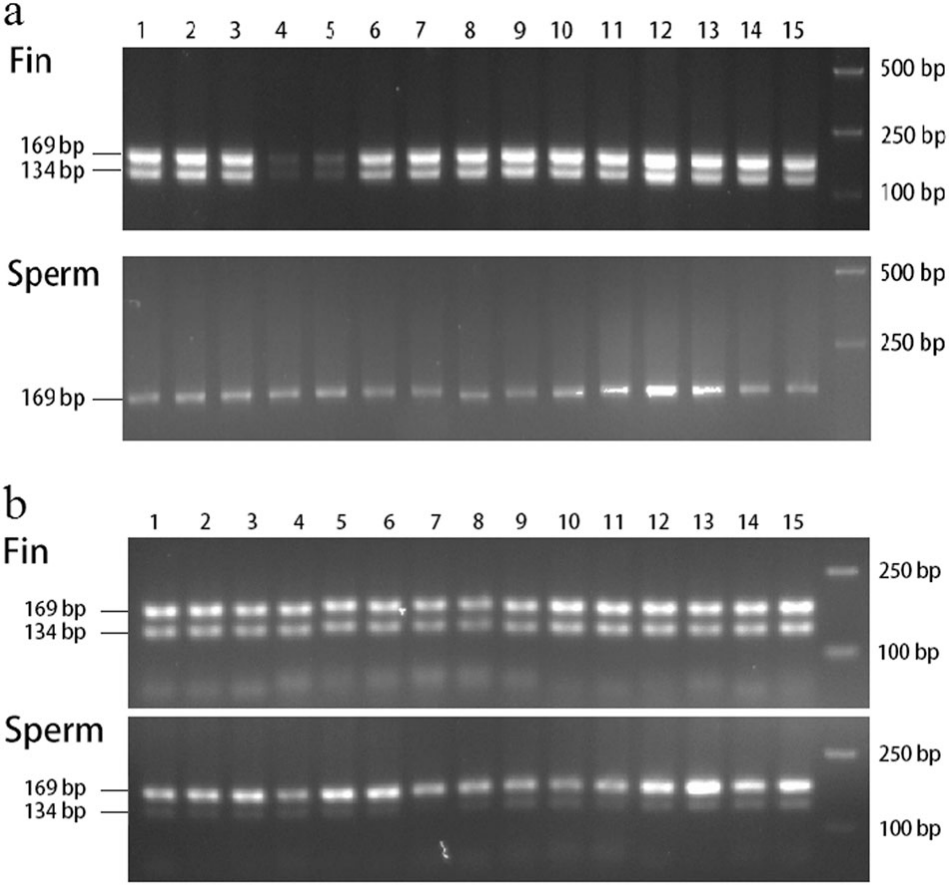
Differences between fin and sperm DNA amplified with sex-specific primers for pseudomales. **a** The 169 and 134 bp bands were Z and W chromosome-specific, respectively. Pseudomales contained both Z and W chromosomes; therefore, the W bands were detected in their fin DNA. The absence of W bands amplified with sperm DNA of a pseudomale showed that it cannot produce W sperm. **b** A replication, although the W band can sometimes be detected in the sperm of a pseudomale, this band was very faint compared with that from the fins. In this context, the faint amplification of the W band depended on the presence of somatic cell DNA in the semen and lysis time during DNA extraction

The semen of tongue sole is usually a mixture of sperm and tissue somatic cells arising from squeezing during milt collection. Hence, we repeated several DNA extractions and PCR reactions for 15 pseudomales. The W bands were usually absent by agarose gel electrophoresis, but they were sometimes faintly visible (Fig. 3); it mainly depended on the somatic cell content in semen and lysis time during DNA extraction. If W sperm was present, the W bands of semen DNA should be as distinct as that of fin DNA. Therefore, we concluded that the pseudomales cannot produce W sperm at all, or at least at levels that are comparable to Z sperm as would be expected if gametogenesis were normal.

## Discussion

Sex reversal is the most important issue in tongue sole aquaculture. In this study, SNP Cyn_Z_8564889 was identified as the second locus strongly associated with sex reversal. It was linked with and close to SNP Cyn_Z_6676874; they simultaneously and interactively regulated sex reversal in the tongue sole. Based on their genetic architecture, some important aspects regarding sex reversal in the Chinese tongue sole can now be explained more clearly.

As can be seen in Table 4, both SNP Cyn_Z_6676874 and Cyn_Z_8564889 played a role in sex reversal. When SNP Cyn_Z_6676874 is detected, in theory, SNP Cyn_Z_8564889 should also be detected readily. However, we did not identify SNP Cyn_Z_8564889 in our previous study (Jiang and Li 2017). We reassessed the 115 samples in our previous study and found that SNP Cyn_Z_8564889 and other SNPs in the confidence interval had not been successfully genotyped. After genotyping the 115 fish for SNP Cyn_Z_8564889 by sequencing, only one individual was found to carry the GG genotype. Therefore, it was impossible to detect SNP Cyn_Z_8564889 with the samples in our previous study because of low detection power and lack of sufficient markers.

In our previous study (Jiang and Li 2017), we found that all the genetic female offspring of a pseudomale would reverse into males, but we did not understand the reasons at that time. Now, this puzzle has been solved with the identification of the new locus in our study and through the pseudomale spermatogenesis validation experiment. A pseudomale, containing both the Z and W chromosomes, was usually regarded as an alternative to improve the female proportion in tongue sole aquaculture. We have confirmed that pseudomales produce mostly or only Z sperm, which had been partially confirmed in our previous study. The offspring of pseudomales included 24 pseudomales (ZW) and 22 normal males (ZZ) (Table 3 in the ref. Jiang and Li 2017), and the ratio of ZW/ZZ was nearly 1. If a pseudomale can produce W sperm, the ratio of ZW/ZZ should be 2/1, because WW individuals cannot survive. However, the ratio was significantly less than 2/1 (*P* = 0.037). The Z chromosome of pseudomales harbors the T allele of SNP Cyn_Z_6676874 and the A allele of SNP Cyn_Z_8564889 simultaneously. Since there is no homologous chromosome for the Z chromosome, it does not experience synapsis during meiosis. These two unfavorable alleles are in linkage and the unfavorable T-A haplotype has no opportunity to separate through crossover and recombination. Therefore, the genetic female offspring of pseudomales are doomed to inherit this unfavorable haplotype and reverse into male generation after generation, expanding very quickly in the population and leading to extremely low percentages of phenotypic females in tongue sole aquaculture. Regarding this issue, using comparative analysis of the gonadal DNA methylomes of pseudomale, female, and normal male fish, Shao et al. (2014) proposed that epigenetic regulation plays multiple crucial roles in the sexual reversal of the tongue sole fish. We believe that the similarity in DNA methylation profiles between pseudomales and normal males may be the result of, and not the reason for, sex reversal; the genetic interaction between the two loci that we found and the absence of W sperm for pseudomales should be the fundamental reasons for sex reversal, and also the possible reason for methylation. The polymorphisms in the two haplotypes (Table 2, Fig. 2) will be translated into different amino acids, which may regulate DNA methylation and the differentiation of sexual gonads. DNA methylation is tissue specific, and very unlikely to have caused DNA mutation.

It is known that pseudomales are the main reason for low female percentages in tongue sole aquaculture. Once a pseudomale is selected for breeding, the female percentages in future generations will decrease significantly. However, it is easier to select a pseudomale over a normal male, not only because pseudomale percentages have increased during the last decade, but also because pseudomales are larger than normal males when mature (Li et al. 2017), although they are smaller than the normal females. It was traditionally thought that larger fathers beget larger children, and breeders preferred larger males for breeding. Recently, some breeders began to examine candidate paternal fish to avoid using pseudomales for breeding, and the percentages of phenotypic females improved, albeit very slowly, because the frequency of favorable alleles of SNP Cyn_Z_8564889 and Cyn_Z_6676874 had become very low owing to the lack of knowledge of the genetic architecture of sex reversal. The genetic interaction between SNP Cyn_Z_8564889 and Cyn_Z_6676874 provides flexible options to select candidate parental fish for breeders. Any single or combined SNP examination can be utilized to improve phenotypic female ratios. Examinations of these two SNPs can also be used to predict sex reversal incidence in the larval stage, which provides the possibility of designing experiments to study sex reversal more intensively.

Before 2017, the genes directly regulating sex differentiation in tongue sole were unknown. *Dmrt1* was once speculated as the critical gene regulating sex differentiation (Chen et al. 2014), but with little direct genetic evidence. Herein, *Dmrt1* was mapped to associate with sex reversal in the tongue sole fish through GWAS, which provided solid evidence for this speculation. By knocking out the *Dmrt1* gene of the genetic male tongue sole, ovary-like testes develop and spermatogenesis is disrupted (Cui et al. 2017). *Dmrt1* was verified as the essential gene of sex differentiation by forward and backward genetic evidence. Similarly, *Dmrt1* was shown to be the primary gene associated with sex reversal in the Chinese soft-shelled turtles (Sun et al. 2017). Sex in both the tongue sole fish and turtles is determined by the ZW chromosome system, and this comparison is perhaps beneficial to understand sex differentiation in the ZW chromosome species. In our study, we found that *Dmrt1* may be duplicated, which has also been observed in other species. Its duplication mode and effect on sex reversal should be studied further in future research.

### Data availability

Supplementary file S1 is a phenotypic file containing id, sire, dam, genetic sex, phenotypic sex, and incidence of sex reversal. Supplementary file S2 contains genotypes (coded as 0, 1, and 2) of the 171 samples, and supplementary file S3 is the SNP information. Data available from the Dryad Digital Repository: 10.5061/dryad.n8h5147.

## Electronic supplementary material

Supplementary files (Fig S1 and Fig S2)
