# Supplementary material for: New locus reveals the genetic architecture of sex reversal in the Chinese tongue sole (Cynoglossus semilaevis)
Source: Heredity (Edinb). 2018 Aug 9;121(4):319–26. doi: 10.1038/s41437-018-0126-6 (PMC6134077; doi:10.1038/s41437-018-0126-6)
Supplement: Supplementary file 1 — Supplementary files (Fig S1 and Fig S2) [file 41437_2018_126_MOESM1_ESM.pdf]

Figure S1: Genotypes of SNP Cyn_Z_8564889. SNP Cyn_Z_8564889 is located in the third intron of the *Dmrt1* gene on the Z chromosome; it has two alleles, A and G (box of dashed lines). Although genetic females contain only one Z chromosome, both alleles A and G can sometimes be detected by sequencing the PCR product, indicating the possible duplication of *Dmrt1*.

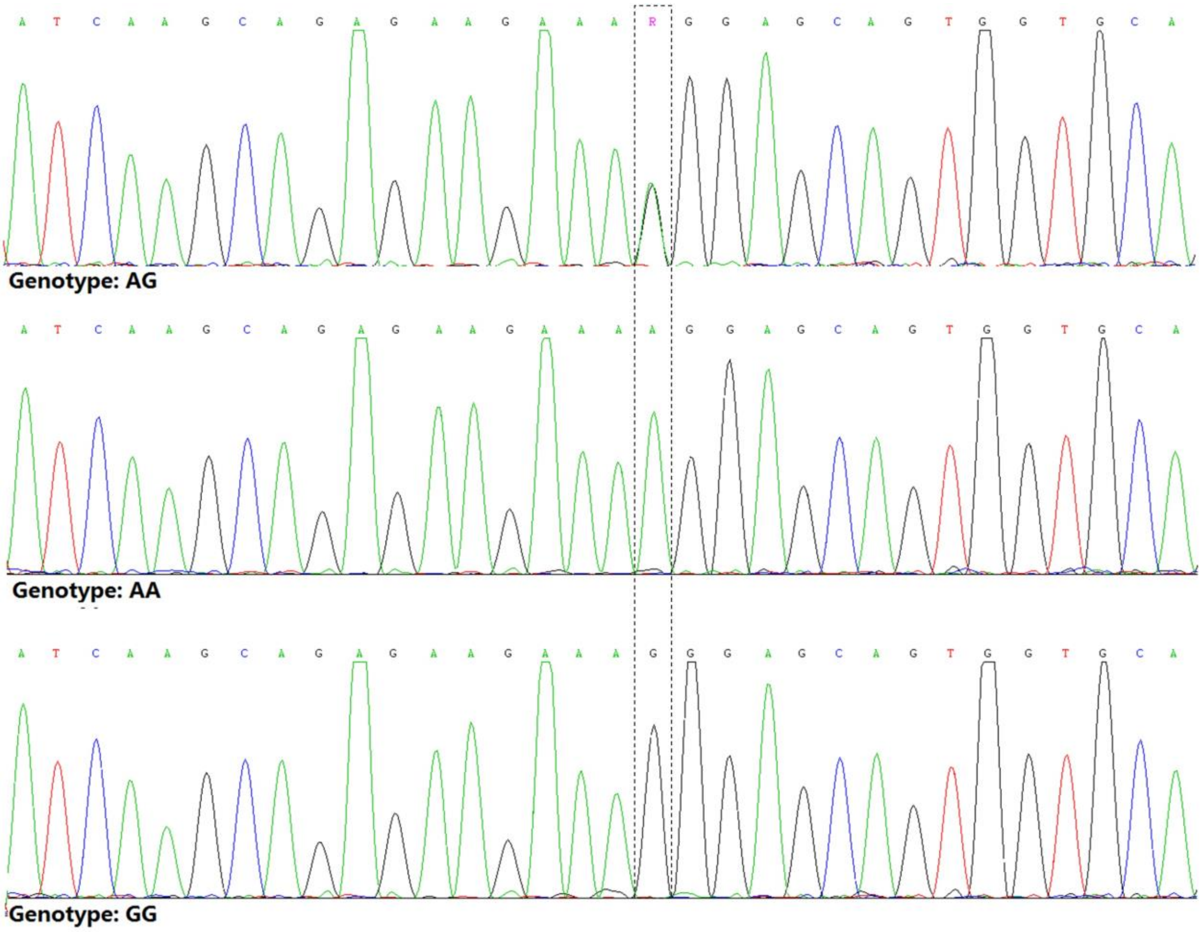

Figure S2: Alignment of the ORF sequences of *Dmrt1* for fish of genotypes AA and GG at SNP Cyn_Z_8564889. The SNP C→A at position 8566940 and SNP C→T at position 8566916 are located in the second exon of *Dmrt1*, which will cause the amino acid changes Ala→Ser and Val→Ile, respectively. The codons and their corresponding amino acids are blackened.

```
GG-ORF. ATG AAC AAG AAC AAG CAG CGC CCT GAC TAC ACT GGA CCA CAG TCC CCA TCC AAA GGC CGA AGA CCA CCC AGG ACG CCC AAG TGC TCC CGG   90
        Met Asn Lys Asn Lys Gln Arg Pro Asp Tyr Thr Gly Pro Gln Ser Pro Ser Lys Gly Arg Arg Pro Pro Arg Thr Pro Lys Cys Ser Arg   30
AA-ORF. ATG AAC AAG AAC AAG CAG CGC CCT GAC TAC ACT GGA CCA CAG TCC CCA TCC AAA GGC CGA AGA CCA CCC AGG ACG CCC AAG TGC TCC CGG   90
        Met Asn Lys Asn Lys Gln Arg Pro Asp Tyr Thr Gly Pro Gln Ser Pro Ser Lys Gly Arg Arg Pro Pro Arg Thr Pro Lys Cys Ser Arg   30

GG-ORF. TGT AGG AAT CAC GGC TTC GTG TCT CCG TTG AAG GGC CAC AAA CGC TAC TGT GAC TGG AGG GAG TGT CGC TGT GAC AAG TGT AAC CTC ATA   180
        Cys Arg Asn His Gly Phe Val Ser Pro Leu Lys Gly His Lys Arg Tyr Cys Asp Trp Arg Glu Cys Arg Cys Asp Lys Cys Asn Leu Ile    60
AA-ORF. TGT AGG AAT CAC GGC TTC GTG TCT CCG TTG AAG GGC CAC AAA CGC TAC TGT GAC TGG AGG GAG TGT CGC TGT GAC AAG TGT AAC CTC ATA   180
        Cys Arg Asn His Gly Phe Val Ser Pro Leu Lys Gly His Lys Arg Tyr Cys Asp Trp Arg Glu Cys Arg Cys Asp Lys Cys Asn Leu Ile    60

GG-ORF. GCG GAG AGA CAG CGA ATC ATG GCG GCG CAG GTT GCC CTG AGG AGG CAG CAG GCC CAG GAG GAA GAA CTT GGG ATT TGT ACT CCA GTT GCT   270
        Ala Glu Arg Gln Arg Ile Met Ala Ala Gln Val Ala Leu Arg Arg Gln Gln Ala Gln Glu Glu Glu Leu Gly Ile Cys Thr Pro Val Ala    90
AA-ORF. GCG GAG AGA CAG CGA ATC ATG GCG GCG CAG GTT GCC CTG AGG AGG CAG CAG GCC CAG GAG GAA GAA CTT GGG ATT TGT ACT CCA GTT TCT   270
        Ala Glu Arg Gln Arg Ile Met Ala Ala Gln Val Ala Leu Arg Arg Gln Gln Ala Gln Glu Glu Glu Leu Gly Ile Cys Thr Pro Val Ser    90

GG-ORF. GTC AAT GGG CCT GAA GTG ATG GTC AAG AGT GAG TCT AGA GCG GAC TGC CTG CTC CCT GTG GAA GGG AGA TCC ATG CCC TCT TCC ATC AGC   360
        Val Asn Gly Pro Glu Val Met Val Lys Ser Glu Ser Arg Ala Asp Cys Leu Leu Pro Val Glu Gly Arg Ser Met Pro Ser Ser Ile Ser   120
AA-ORF. GTC AAT GGG CCT GAA GTG ATG ATC AAG AGT GAG TCT AGA GCG GAC TGC CTG CTC CCT GTG GAA GGG AGA TCC ATG CCC TCT TCC ATC AGC   360
        Val Asn Gly Pro Glu Val Met Ile Lys Ser Glu Ser Arg Ala Asp Cys Leu Leu Pro Val Glu Gly Arg Ser Met Pro Ser Ser Ile Ser   120

GG-ORF. ACC TCC ACT TAT GTG CAT GCT GGC CAA GGG AGC AGC AGG GCT CAT CAT GAG GGA TCG TCT GAC CTT CAG ATG GAA ACC CCC TAT TAC AAC   450
        Thr Ser Thr Tyr Val His Ala Gly Gln Gly Ser Ser Arg Ala His His Glu Gly Ser Ser Asp Leu Gln Met Glu Thr Pro Tyr Tyr Asn   150
AA-ORF. ACC TCC ACT TAT GTG CAT GCT GGC CAA GGG AGC AGC AGG GCT CAT CAT GAG GGA TCG TCT GAC CTT CAG ATG GAA ACC CCC TAT TAC AAC   450
        Thr Ser Thr Tyr Val His Ala Gly Gln Gly Ser Ser Arg Ala His His Glu Gly Ser Ser Asp Leu Gln Met Glu Thr Pro Tyr Tyr Asn   150

GG-ORF. ATC TAC CAA CCA TCT CGT TAC CTG TAC AAC TAT CAG CAA TAC CAG ATG TCT CAT GGT GAT GGC TGC CTG CCG AGC CAC AAC ATG CCC TCT   540
        Ile Tyr Gln Pro Ser Arg Tyr Leu Tyr Asn Tyr Gln Gln Tyr Gln Met Ser His Gly Asp Gly Cys Leu Pro Ser His Asn Met Pro Ser   180
AA-ORF. ATC TAC CAA CCA TCT CGT TAC CTG TAC AAC TAT CAG CAA TAC CAG ATG TCT CAT GGT GAT GGC TGC CTG CCG AGC CAC AAC ATG CCC TCT   540
        Ile Tyr Gln Pro Ser Arg Tyr Leu Tyr Asn Tyr Gln Gln Tyr Gln Met Ser His Gly Asp Gly Cys Leu Pro Ser His Asn Met Pro Ser   180

GG-ORF. CAG TAC TGC ATG CAT TCT TAC TAC CCA GCA ACC TCC TAC CTG ACC CAA GGC CGC AGC TCT GCC ACC TAC GTT CCT TCC ATC TGC AAC CTG   630
        Gln Tyr Cys Met His Ser Tyr Tyr Pro Ala Thr Ser Tyr Leu Thr Gln Gly Arg Ser Ser Ala Thr Tyr Val Pro Ser Ile Cys Asn Leu   210
AA-ORF. CAG TAC TGC ATG CAT TCT TAC TAC CCA GCA ACC TCC TAC CTG ACC CAA GGC CGC AGC TCT GCC ACC TAC GTT CCT TCC ATC TGC AAC CTG   630
        Gln Tyr Cys Met His Ser Tyr Tyr Pro Ala Thr Ser Tyr Leu Thr Gln Gly Arg Ser Ser Ala Thr Tyr Val Pro Ser Ile Cys Asn Leu   210

GG-ORF. GAG GAC GGC AAC TAC GGC AGT AAC AAC AAC TAC GCC GAG ACC ACG GCA GCC TCC GCC TCG TCC AGC GTC GGC CTC ACC GCC GCT CCT GAC   720
        Glu Asp Gly Asn Tyr Gly Ser Asn Asn Asn Tyr Ala Glu Thr Thr Ala Ala Ser Ala Ser Ser Ser Val Gly Leu Thr Ala Ala Pro Asp   240
AA-ORF. GAG GAC GGC AAC TAC GGC AGT AAC AAC AAC TAC GCC GAG ACC ACG GCA GCC TCC GCC TCG TCC AGC GTC GGC CTC ACC GCC GCT CCT GAC   720
        Glu Asp Gly Asn Tyr Gly Ser Asn Asn Asn Tyr Ala Glu Thr Thr Ala Ala Ser Ala Ser Ser Ser Val Gly Leu Thr Ala Ala Pro Asp   240

GG-ORF. TTT GCC CTG AAC TAC ACC GTC ACC TCC ATC GTT TAC GGT GAA ACA AAC AAA TAA                                                  774
        Phe Ala Leu Asn Tyr Thr Val Thr Ser Ile Val Tyr Gly Glu Thr Asn Lys ter                                                  258
AA-ORF. TTT GCC CTG AAC TAC ACC GTC ACC TCC ATC GTT TAC GGT GAA ACA AAC AAA TAA                                                  774
        Phe Ala Leu Asn Tyr Thr Val Thr Ser Ile Val Tyr Gly Glu Thr Asn Lys ter                                                  258
```
